# Recombinant NAD-dependent SIR-2 Protein of *Leishmania donovani*: Immunobiochemical Characterization as a Potential Vaccine against Visceral Leishmaniasis

**DOI:** 10.1371/journal.pntd.0003557

**Published:** 2015-03-06

**Authors:** Rajendra K Baharia, Rati Tandon, Tanuj Sharma, Manish K Suthar, Sanchita Das, Mohammad Imran Siddiqi, Jitendra Kumar Saxena, Shyam Sunder, Anuradha Dube

**Affiliations:** 1 Division of Parasitology, CSIR-Central Drug Research Institute, Lucknow, India; 2 Division of Molecular and Structural Biology, CSIR-Central Drug Research Institute, Lucknow, India; 3 Division of Biochemistry, CSIR-Central Drug Research Institute, Lucknow, India; 4 Department of Medicine, Institute of Medical Sciences, Banaras Hindu University, Varanasi, India; Universidade Federal de Minas Gerais, BRAZIL

## Abstract

**Background:**

The development of a vaccine conferring long-lasting immunity remains a challenge against visceral leishmaniasis (VL). Immunoproteomic characterization of *Leishmania donovani* proteins led to the identification of a novel protein NAD+-dependent Silent Information regulatory-2 (SIR2 family or sirtuin) protein (LdSir2RP) as one of the potent immunostimulatory proteins. Proteins of the SIR2 family are characterized by a conserved catalytic domain that exerts unique NAD-dependent deacetylase activity. In the present study, an immunobiochemical characterization of LdSir2RP and further evaluation of its immunogenicity and prophylactic potential was done to assess for its possible involvement as a vaccine candidate against leishmaniasis.

**Methodology/Principal Findings:**

LdSir2RP was successfully cloned, expressed and purified. The gene was present as a monomeric protein of ~45 kDa and further established by the crosslinking experiment. rLdSir2RP shown cytosolic localization in *L*. *donovani* and demonstrating NAD+-dependent deacetylase activity. Bioinformatic analysis also confirmed that LdSir2RP protein has NAD binding domain. The rLdSir2RP was further assessed for its cellular response by lymphoproliferative assay and cytokine ELISA in cured *Leishmania* patients and hamsters (*Mesocricetus auratus*) in comparison to soluble *Leishmania* antigen and it was observed to stimulate the production of IFN-γ, IL-12 and TNF-α significantly but not the IL-4 and IL-10. The naïve hamsters when vaccinated with rLdSir2RP alongwith BCG resisted the *L*. *donovani* challenge to the tune of ~75% and generated strong IL-12 and IFN-γ mediated Th1 type immune response thereof. The efficacy was further supported by remarkable increase in IgG2 antibody level which is indicative of Th1 type of protective response. Further, with a possible implication in vaccine design against VL, identification of potential T-cell epitopes of rLdSir2RP was done using computational approach.

**Conclusion/Significance:**

The immunobiochemical characterization strongly suggest the potential of rLdSir2RP as vaccine candidate against VL and supports the concept of its being effective T-cell stimulatory antigen.

## Author Summary

Visceral Leishmaniasis (VL) is the most fatal form of leishmaniasis disease in Indian subcontinent. Through proteomic approaches, NAD-dependent Silent information regulator-2 was identified as one of the potent immunostimulatory proteins. Herein, it was first reported the cloning, expression, purification and immunobiochemical characterization of a NAD+-dependent protein from *Leishmania donovani*. The gene encodes a monomeric protein (LdSir2RP) of approximately 45 kDa and showed NAD+-dependent deacetylase activity. LdSir2RP was immunodetected in whole cell lysate of *L*. *donovani* and further it immunolocalized in cytoplasm of the *Leishmania* parasite. Recombinant protein rLdSir2RP shown immunogenicity in PBMCs of cured *Leishmania* patients and hamsters (*Mesocricetus auratus*). rLdSir2RP stimulated the production of IFN-γ, IL-12 and TNF-α but not IL-4 and IL-10. This was further supported by remarkable increase in IgG2 antibody level.

It was further demonstrated that rLdSir2RP was able to provide considerable protection to hamsters against *L*. *donovani* challenge. These results supported by the increased iNOS mRNA transcript and the specific Th1-type cytokines—IFN-γ, IL-12 and TNF-α and down-regulation of IL-4, IL-10 and TGF-β. Hence, it is inferred that rLdSir2RP confer significant protection against experimental VL and considered as potential vaccine targets against visceral leishmaniasis.

## Introduction

Leishmaniases (cutaneous, mucocutaneous, and visceral) is caused by an intracellular protozoan parasite complex by the invasion of the reticuloendothelial system. Among the three types of clinical manifestations visceral leishmaniasis (VL) disease is the most severe one and remains a major concerned public health problem in tropical and subtropical countries. VL is a systemic disease and is characterized by intermittent fever, hepatosplenomegaly, cachexia, pancytopenia, and hypergammaglobulinemia [1]. The disease is common in less developed countries [2] including Indian subcontinent and is highly prevalent in the North-Eastern states of India particularly Bihar, Assam, West Bengal and eastern Uttar Pradesh [3,4]. Recent epidemics of VL in Sudan and India have resulted in over 100,000 deaths [3]. During the last 20 years the spectrum of Leishmaniasis has modified, due to the development of the HIV/AIDS [4]. Moreover, the available antileishmanial drugs are very costly and having long-term course of treatment with adverse side effects. Apart from this in the various region of endemicity the increasing drug resistance has worsened the scenario. This situation necessitates opting for an alternative strategy and therefore, development of a vaccine would be a better option for an effective control strategy for VL. In active VL cases cell-mediated immune responses are absent [5–7] and in the patients that are cured, the Th1 type immune response is increased [8–10] leading to long time immunity. This provides a rationale that Th1 immune response play a major role in cure and prevention of VL [7] Therefore, the antigenic proteins that modulate Th1 type arm of the immune response could be exploited as vaccine candidates.

*Leishmania donovani* NAD-dependent Silent information regulator protein-2 (LdSir2RP) was identified as one of the Th1 stimulatory protein through immunoproteomics from soluble leishmanial lysate [11]. Silent information regulator 2 (Sir2) proteins, or sirtuins, are protein deacetylases dependent on nicotine adenine dinucleotide (NAD) and are found in organisms ranging from bacteria to humans. The importance of NAD+-dependent deacetylases (Sir2 family or Sirtuins) has been reported in cell survival, ageing and apoptosis and has led to chemical and cellular investigations aimed at understanding this unique class of enzymes.

SIR2 proteins have been characterized from *L*. *major* (LmSIR2) [12] and *L*. *infantum* (LiSIR2) [13] while two other related sequences can be found in the *Leishmania* genome database (*L*. *major* sirtuin (CAB55543), *L*. *major* cobB (LmjF34.2140). Using indirect molecular and biological approaches Vergnes *et al*. [14] showed the potential role of LmSIR2 and LiSIR2 genes in parasite survival, due to an inherent resistance to apoptosis-like death [13]. This led to the generation of a SIR2-deficient *L*. *infantum* mutant cell line which was found to be protective against homologous challenge [14]. The role of Sir 2 protein has not been so far explored in *L*. *donovani* a severe and fatal form of VL widely prevalent in Sudan and Indian subcontinent. In the present study, molecular and immunobiochemical characterization of LdSir2RP was carried out and explored its function using bioinformatics approaches. The ability of recombinant rLdSir2RP to stimulate the immune responses in *Leishmania* infected cured /endemic contact individuals’ PBMCs and to protect naive hamsters against *L*. *donovani* challenge was further examined.

## Materials and Methods

### Animals, parasites and cell line culture

Laboratory-bred male golden hamsters (*Mesocricetus auratus*, 45–50 g) from the Institute’s animal house facility were used as experimental host. They were housed in a climatically controlled room and fed with standard rodent food pellet (Lipton India, Mumbai, India) and water *ad libitum*. The *L*. *donovani* WHO reference strain Dd8 (MHOM/In/80/Dd8) was cultured *in vitro* as described elsewhere [15]. The strain has also been maintained in hamsters through serial passage, i.e. from amastigote to amastigote [16]. Mouse macrophage cell line J774A.1 was procured from Tissue Culture Facility of the Central Drug Research institute, Lucknow and maintained in RPMI-1640 at 37°C and 5% CO_2_. The confluent cells were harvested using cell scrapper for the estimation of nitric oxide (NO) production.

### Soluble antigen of *L*. *donovani* (SLD) promastigote

Soluble *L*. *donovani* (SLD) promastigote antigen was prepared as per method described by Gupta *et al*. [11]. The Log phase promastigotes (10^9^) were harvested from culture and washed 3 to 4 times in cold PBS, resuspended in PBS containing protease inhibitors cocktail (Sigma, USA) and subjected to ultrasonication and centrifugation at 40,000×g for 30min. The protein content of the supernatant was estimated [17] and stored at −70°C.

### Cloning, expression and purification of recombinant NAD-dependent Silent information regulator protein—(rLdSir2RP)

*L*. *donovani* genomic DNA was isolated from 10^8^ promastigotes and extracted by phenol: chloroform: isoamyl alcohol extraction and ethanol precipitation. LdSir2RP gene was amplified using LdSir2RP specific primers (based on the *L*. *infantum*—LiSir2RP gene sequence): forward, 5’-GGATCCGAGAAGGGCACTGTGGCGATC-3’ and reverse, 5’-GAATTCTTACAACTTATCCATGAACTGGTC-3’ (*BamHI* and *EcoRI*) (Fermentas) site underlined) in a Thermocycler (Mini Bio-Rad) under conditions at one cycle of 95°C for 4 min, 30 cycles of 95°C for 1min, 55°C for 1min, and 72°C for 2 min, and finally one incubation of 72°C for 10 min. Amplified PCR product was ligated in pTZ57R/T (T/A) cloning vector (Fermentas) and transformed into competent *Escherichia coli DH5α* cells. The LdSir2RP insert was screened by gene-specific PCR under similar conditions as previously mentioned. Isolated positive clones were sequenced from Delhi University (New Delhi, India) and submitted to the National Center for Biotechnology Information (http://www.ncbi.nlm.nih.gov/nuccore/JN790591.1); accession no. JN790591.1). LdSir2RP was further subcloned at the *BamHI* and *EcoRI* site in bacterial expression vector pET28a^+^ (Novagen). The transformed cells were inoculated into 5 ml Luria-Bertani medium (LB) containing 34 μg/mL of chloramphenicol and 35 μg/mL kanamycin and allowed to grow at 37°C in a shaker at 200 rpm. Cultures in logarithmic phase (at OD_600_ of ~ 0.5–0.6) were induced for 3h with 1.0 mM isopropyl-b-D-thiogalactopyranoside (IPTG) (Sigma) at 37°C. After induction, 1 ml cells were lysed in 100μl sample buffer (50m M Tris-HCl (pH 8), 10% SDS, and 0.05% bromophenol blue, with 100 mM DTT) and whole cell lysates (WCL) were analyzed by 12% SDS-PAGE. Uninduced control culture was analyzed in parallel. The over expression of rLdSir2RP was visualized by staining the gel with Coomassie brilliant blue R-250 (Sigma).

For purification 200 mL of LB medium were inoculated with *E*.*coli rosetta* strain transformed with pET28a+LdSir2RP, and grown at 37°C to an O.D._600_ of ~ 0.6. The rLdSir2RP was purified by affinity chromatography using Ni^2+^chelating resin to bind the His6-tag fusion peptide derived from the pET28a^+^ vector. The cell pellet was resuspended in 4 mL of lysis buffer (50 mM Tris-HCL (pH 8.0), 300 mM NaCl) containing 1:200 dilution of protease cocktail inhibitor (Sigma) and 1% Triton X-100, incubated for 30 min on ice with 1 mg/mL lysozyme (Sigma). Recombinant rLdSir2RP was eluted with elution buffer (50 mM Tris-HCL, 100 mM NaCl, and 250 mM imidazole, pH 8.0). The eluted fractions were analyzed by 12% SDS—PAGE for purity and stained. The protein content of the fractions was estimated by the Bradford method using BSA as standard.

### Polyclonal antibodies produce against rLdSir2RP and western blot analysis

The purified rLdSir2RP protein was used for raising antibodies in swiss mice. Mice were first immunized using 50 μg of rLdSir2RP in Freund’s complete adjuvant (Merck). After 15 days, the mice was given 3 booster doses of 25μg rLdSir2RP each in incomplete Freund’s adjuvant (Merck) at 2-weeks interval and blood was collected for serum 8 days after the last immunization. Antibody titre was determined by ELISA and was found to be 1:64000. For immunoblotting experiment, purified rLdSir2RP protein and SLD were resolved on 12% SDS—PAGE and transferred on to nitrocellulose membrane using a semi-dry blot apparatus (Amersham) [18]. After overnight blocking in 5% BSA, the membrane was incubated with antiserum to the rLdSir2RP protein at a dilution of 1:2000 for 120 min at room temperature (RT). The membrane was washed three times with PBS containing 0.5% Tween 20 (PBS-T) and then incubated with goat anti-mice IgG HRP conjugate (Bangalore Genie) at a dilution of 1:10,000 for 1h at RT. Blot was developed by using diaminobenzidinehydrochloride + imidazole +H_2_O_2_ (Sigma).

### Immunolocalization of rLdsir2RP in *Leishmania* parasite with fluorescence microscopy

*L*. *donovani* promastigotes were washed in PBS, fixed with formaldehyde and allowed to adhere to poly-(L-lysine)-coated coverslips. Cells were then permeabilized with 0.2% (v/v) Triton X-100 and washed again with PBS. The coverslips with fixed cells were incubated in PBS containing 3% BSA for 30min and washed again with PBS. Then, the cells were incubated with primary antibodies (mice anti-*L*. *donovani* rLdSir2RP) at a dilution 1:2000 for 1h, rinsed with PBS and incubated for 1h with FITC conjugated goat anti-mice IgG. The coverslips were mounted on clean slides using 10 μl of mounting media (Calibiochem) and finally the slides were observed under confocal laser scanning microscope (Zeiss LSM 510 Meta) under a 60×oil immersion plan apochromate objective lens (NA 1.4). The negative control samples were processed in parallel by omitting primary antibodies that were used to negate background fluorescence, if any, by adjusting laser power and gain/offset settings before image acquisition. Images were processed in Adobe Photoshop (version 7.1) for presentation purposes.

### Bioinformatics analysis

Given seven sequences were aligned by using clustal W2 [19, 20] software at EBI. MSA file was generated in PHYLIP format and file so generated was then opened in Mega 5.2 tool [21, 22]. File was then converted to MEGA file format and using MEGA file format phylogenetic tree was built using MEGA 5.2 tool, using Maximum Parsimony approach [23]. Tool has been previous cited in other research articles [24–26] for constructing Phylogenetic tree. Given sequences were then uploaded in GLAM2 tool [27] of MEME suite [28] to identify motifs present in group of related protein sequences. This regular expression was queried in CDART tool [29] to identify the functionality of motif. Same results were confirmed by Conserved Domain Database [30] and SMART tool [31].

### Mapping of T-cell epitopes using bioinformatics

Immune epitope database (IEDB) server (www.iedb.org) was used for prediction of potential promiscuous T-cell epitopes on NAD sequence [32]. The IEDB uses information related to all experimentally determined immune epitope. It uses ANN, SMM, SMMPMBEC, CombLib Consensus, and NetMHCpan approach to predict the potential epitopes on user submitted protein sequence [33–37]. It covered wide range of MHC alleles. Both MHC-I and MHC-II based peptides were predicted. Server has been used for epitope prediction previously [38–40]. For prediction, we considered MHC alleles most prevalent in north Indian population [41].

### Glutaraldehyde cross-linking studies and its deacetylase activity assay

Glutaraldehyde cross-linking analysis with purified native SiR2 was carried out as described earlier [42]. To native SiR2 (100 μg/ml) was added an aliquot of 25% (v/v) glutaraldehyde (Sigma) to achieve a final concentration of 1% (v/v) glutaraldehyde and incubated at 25°C for 5min. Cross linking sample was quenched by addition of 200mM sodium borohydride. After 20min incubation, 3μl of 10% (w/v) aqueous sodium deoxycholate was added. The pH of the reaction mixture was decreased to 2–2.5 by the addition of orthophosphoric acid that resulted in precipitation of the cross-linked SiR2 protein. After centrifugation (13,000×g, 4°C, 10 min) the obtained precipitates were re-dissolved in 0.1M Tris—HCl (pH 8.0), 1% SDS (w/v) and 50mM DTT and heated at 90–100°C. The molecular mass of the crosslinked products were determined by 6% (w/v) SDS—PAGE. Enzyme activity for SIR2 deacetylase was determined fluromatric using CycLex SIR2 deacetylase assay kit with some modifications [43]. Briefly enzyme reaction was performed in a 96 well plate consisting fluorescence-labelled acetylated peptide as substrate for deacetylase, NAD and lysylendopeptidase in buffer (Tris-HCl, pH 8.8). Reaction was initiated by adding purified recombinant SIR2 deacetylase protein. Fluorescence was measured with excitation at 490 nm and emission at 530 nm.

### Patients and isolation of PBMCs

The study groups for human samples were as follows:

1. Eight endemic household contacts (2 males and 6 females, age range-25 to 55 years) who neither showed clinical symptoms nor received any treatment for Kala-azar. They belonged to the family of infected/cured patients.
2. Eight treated cured patients (2 males and 6 females, age ranging from 7–40 years) from hyper-endemic areas of Bihar. All the patients had received complete course of amphotericin B and had recovered from VL. Samples were collected from 2 months to 1 year after the completion of treatment. Diagnosis was established in all cases by demonstration of parasites in splenic aspirates and found negative at the time of study.
3. Eight infected patients (5 males and 3 females, age range- 15 to 50 years) showing clinical symptoms of Kala-azar.
4. Eight normal healthy donors (6 males and 2 females, age range 25–35 years) from non-endemic areas, without any history of leishmaniasis, served as negative control. The study was approved by the Ethics committee of the Kala-azar Medical Research Centre, Muzaffarpur (Protocol # EC-KAMRC/Vaccine/VL/2007–01).

Peripheral blood mononuclear cells (PBMCs) were isolated from blood by Ficoll-Hypaque density gradient centrifugation (Histopaque 1077, Sigma, USA) as described by Garg *et al*. [15]. A final suspension of 1×10^6^ cells/ml was made in complete RPMI medium (cRPMI) after determining cell viability by trypan blue staining method. These were used for various immunological assays.

### Treatment of *L*. *donovani* infected hamsters and isolation of mononuclear cells (lymph node cells)

Golden hamsters (*Mesocricetus auratus*, 45–50 g) from the Institute’s animal house facility were used as experimental host. Approximately 20 hamsters, infected with 10^7^ amastigotes intracardially, were assessed one month later for parasitic burden by splenic biopsy through a small incision in the upper left quarter of the abdomen and a small piece of splenic tissue was cut and dab smears were made on slides. The smears were fixed in methanol, stained with Giemsa and the number of amastigotes/1000 cell nuclei was counted. The animals harbouring >25–30 amastigotes/100 macrophage cell nuclei were then treated with antileishmanial drug-Miltefosine (Zentaris, Germany) at 40 mg/kg bodyweight daily for 5 days. The animals were reassessed for complete cure by splenic biopsy performed on day 30 post-treatment. Mononuclear cells were separated from lymph nodes of cured, infected as well as normal hamsters following the protocol of Garg *et al*. [15] and a suspension of 10^6^ cells/ml was made in cRPMI. These cells were employed for lymphoproliferative assay and for the estimation of NO production.

### Assessment of lymphocyte proliferative responses (LTT) in cured/exposed patients, hamsters and nitric oxide production

Lymphocytes suspension (1×10^6^ cells/ml) of cured/exposed patients and normal, infected (30 days p.i.) and cured hamsters was cultured in 96-well flat bottom tissue culture plates (corning). This assay was carried out as per protocol described by Garg *et al*. [15] with some modifications, wherein XTT (Roche diagnostics) was used instead of ^3^H thymidine. About 100μl of predetermined concentration (10μg/ml) of mitogens (PHA for Patient’s PBMCs; Con A for hamster’s lymphocytes), as well as rLdSir2RP and SLD (10μg/ml each) were added to the wells in triplicate. Wells without stimulants served as blank controls. Cultures were incubated at 37°C in a CO_2_ incubator with 5% CO_2_ for 3 days in the case of the mitogens, and for 5 days in the case of the antigens. Eighteen h prior to termination of culture, 50 μl of XTT was added to 100 μl of supernatants of each well and absorbance measured at 480 nm with 650 nm as reference wavelength. Isolated lymphocytes from all the three study groups of hamsters viz. Normal, infected (30 p.i.) and cured, were suspended in culture medium and plated at 10^5^ cells/well and stimulated for 3 days in case of mitogen (LPS) and 5 days in case of antigens (rLdSir2RP, SLD) at 10 μg /ml. The presence of NO was assessed using Griess reagent (Sigma, U.S.A) in the culture supernatants of [44] macrophage cell lines (J774 A.1) after the exposure with supernatant of stimulated lymphocyte’s. The supernatants (100 μl) collected from macrophage cultures 24 h after incubation was mixed with an equal volume of Griess reagent and left for 10 min at room temperature. The absorbance of the reaction was measured at 540 nm in an ELISA reader [44]. The nitrite concentration in the macrophages culture supernatant samples was extrapolated from the standard curve plotted with sodium nitrite.

### Assessment of cytokine levels in lymphocytes of cured/endemic patients

Culture of PBMCs (1×10^6^ cells/ml) from human patients was set up in 96-well culture plates and rLdSir2RP was added at a concentration of 10 μg/ml in triplicate wells. The level of IFN-γ, IL-12 as well as IL-10 was estimated by ELISA kit (OptEIA set, Pharmingen) after 5 days of incubation with antigens using supernatants. The results were expressed as picograms of cytokine/ml, based on the standard curves of the respective cytokine provided in the kit.

### Determination of antibody response in hamsters

The level of IgG antibody and its isotypes in sera samples of hamsters of different experimental groups was measured as per protocol by Samant *et al*. [45] with slight modifications. Briefly, 96-well ELISA plates (corning) were coated with rLdSir2RP (0.2 μg/100 μl/well) overnight at 4°C and blocked with 1.5% BSA at room temperature for 1h. Sera was used at a dilution of 1/100 for IgG, IgG1, and IgG2 and kept for 2h at RT. Biotin-conjugated mouse anti-Armenian and Syrian hamster IgG, IgG1 and biotinylated anti-Syrian hamster IgG2 (BD Pharmingen) were added for 1h at room temperature at 1/1000 dilutions and were further incubated with peroxidase-conjugated streptavidin at 1/1000 (BD Pharmingen) for 1h. Finally, the substrate O-phenylenediamine dihydrochloride (Sigma) was added and the plate was read at 492 nm.

### Prophylactic efficacy of recombinant rLdSir2RP protein with or without BCG

Four groups of hamsters (12–15 per group), were taken for the study, wherein groups 1–3 served as controls as described below and group 4 as the main experimental group: group 1, unvaccinated and unchallenged (normal control); group 2, unvaccinated and challenged (infected control); group 3, BCG-0.1mg (Tubervac-Bharat serums and vaccine Limited) alone; and group 4, vaccinated with rLdSir2RP (vaccinated group). The hamsters of Group 4 were immunized intradermally on the back with recombinant rLdSir2RP protein (50μg/50 μl per animal) along with equal volume of BCG 0.1mg per animal in emulsified form and group 3 was given BCG only. Fifteen days later a booster dose of half of the amount of recombinant rLdSir2RP along with BCG was given intradermally to all the hamsters Group 4 and only BCG to group 3. Groups 2–4 were challenged intracardially with 10^7^ metacyclic promastigotes of *L*. *donovani* twenty one days later. On days 45, 60, 90 p.c, 3–4 animals from each group were sacrificed and weights of body, liver and spleen were recorded as well as blood was collected for sera and lymph nodes were removed. Dab smears of liver and spleen were prepared and examined microscopically to estimate parasite burden which was calculated as the number of amastigotes per 100 nucleated cells. The percentage of inhibition of parasite multiplication was calculated in comparison with the unvaccinated control using the following formula: percentage of inhibition = (number of parasite count from infected control—number of parasites from the vaccinated group/number of parasite count from infected control) ×100. Immunological assessment was done (cytokines by real-time PCR and antibody level by ELISA and lymphoproliferation and NO production by above mention methods). Animal of all groups were given proper care and observed for their survival period.

### Quantification of mRNA cytokines and inducible NO synthase (iNOS) in hamsters by real time-PCR & Antibody response in vaccinated/immunized hamsters

QRT-PCR was performed to assess the expression of mRNAs for various cytokines and iNOS in splenic cells. Splenic tissues were taken from each of the three randomly chosen animals. Total RNA was isolated using Tri-reagent (Sigma-Aldrich) and quantified by using Gene-quant (Bio-Rad). One microgram of total RNA was used for the synthesis of cDNA using a first-strand cDNA synthesis kit (Fermentas). For real-time PCR, primers were designed using Beacon Designer software (Bio-Rad) on the basis of cytokines and iNOS mRNA sequences available on PubMed (Table 1). qRT-PCR was conducted as per the protocol described earlier [46] by using 12.5 μl of SYBR green PCR master mix (Bio-Rad), 1 μg of cDNA, and primers at a final concentration of 300 nM in a final volume of 25 μl. PCR was conducted under the following conditions: initial denaturation at 95°C for 2 min followed by 40 cycles, each consisting of denaturation at 95°C for 30 s, annealing at 55°C for 40 s, and extension at 72°C for 40 s per cycle using the iQ5 multicolor real-time PCR system (Bio-Rad). cDNAs from normal hamsters were used as “comparator samples” for quantification of those corresponding to test samples whereas in vaccination studies, cDNAs from infected hamsters were used as “comparator samples”. All quantifications were normalized to the housekeeping gene HPRT. A no-template control c-DNA was included to eliminate contaminations or nonspecific reactions. The cycle threshold (CT) value was defined as the number of PCR cycles required for the fluorescence signal to exceed the detection threshold value (background noise). Differences in gene expression were calculated by the comparative CT method [46]. This method compares test samples to a comparator sample and uses results obtained with a uniformly expressed control gene (HPRT) to correct for differences in the amounts of RNA present in the two samples being compared to generate a ΔCT value. Results are expressed as the degrees of difference between ΔCT values of test and comparator samples. The level of IgG antibody and its isotypes in sera samples of hamsters of different experimental groups was measured as per protocol by Samant *et al*. [45] with slight modifications. Briefly, 96-well ELISA plates (corning) were coated with rLdSir2RP (0.2 μg/100 μl/well) overnight at 4°C and blocked with 1.5% BSA at room temperature for 1 h. Sera was used at a dilution of 1/100 for IgG, IgG1, and IgG2 and kept for 2h at RT. Biotin-conjugated mouse anti-Armenian and Syrian hamster IgG, IgG1 and biotinylated anti-Syrian hamster IgG2 (BD Pharmingen) were added for 1h at room temperature at 1/1000 dilutions and were further incubated with peroxidase-conjugated streptavidin at 1/1000 (BD Pharmingen) for 1 h. Finally, the substrate O-phenylenediamine dihydrochloride (Sigma) was added and the plate was read at 492 nm.

**Table 1:** Sequences of forward and reverse primers of hamster cytokines used for quantitative real time RT-PCR.

S.N.	Primer	Primer sequence
1	HGPRT	Forward5’ GATAGATCCACTCCCATAACTG 3’
		Reverse 5’ TACCTTCAACAATCAAGACATTC 3’
2	TNF-α	Forward 5’ TTCTCCTTCCTGCTTGTG3’
		Reverse 5’ CTGAGTGTGAGTGTCTGG3’
3	IFN-γ	Forward 5’ GCTTAGATGTCGTGAATGG 3’
		Reverse 5’ GCTGCTGTTGAAGAAGTTAG 3’
4	IL-12	Forward 5’ TATGTTGTAGAGGTGGACTG3’
		Reverse 5’ TTGTGGCAGGTGTATTGG 3’
5	TGF-β	Forward 5’ ACGGAGAAGAACTGCTGTG 3’
		Reverse 5’ GGTTGTGTTGGTTGTAGAGG 3’
6	IL-4	Forward 5’ GCCATCCTGCTCTGCCTTC 3’
		Reverse 5’ TCCGTGGAGTTCTTCCTTGC 3’
7	IL-10	Forward 5’ TGCCAAACCTTATCAGAAATG3’
		Reverse 5’ AGTTATCCTTCACCTGTTCC 3’
8	iNOS	Forward 5’ CGACGGCACCATCAGAGG 3’
		Reverse 5’AGGATCAGAGGCAGCACATC 3’

### Ethics statement

Experiments on the animals (hamsters) were performed following the approval of the protocol and the guidelines of Institutional Animal Ethics Committee (IAEC) of the CDRI which is adhered to National Guideline of CPCSEA (Committee for the Purpose of Control and Supervision on Experiments on Animals) under the Ministry of Environment and Forest, Government of India. The approval reference number 154/10/Para/IAEC/2011.

The protocol and study with patients’ was approved by the Ethics committee of the Kala-azar Medical Research Centre, Muzaffarpur (Protocol # ECKAMRC/Vaccine/VL/2007–01) and written informed consent was obtained from patients before enrolment to this study. All the human subjects underwent clinical examination by a local physician for *Leishmania*l and other possible infections.

### Statistical analysis

Results were expressed as mean±S.D. Two sets of experiments were performed and the results were analyzed by student t test and one-way ANOVA test followed by Dunnets or Tukeys post which ever appropriate at each case using Prism Graphpad software program. The upper level of significance was chosen as p<0.001 (highly significant).

## Results

### rLdSir2RP was cloned, sequenced, expressed in *E*. *coli Rosetta* strain

**Fig 1:**
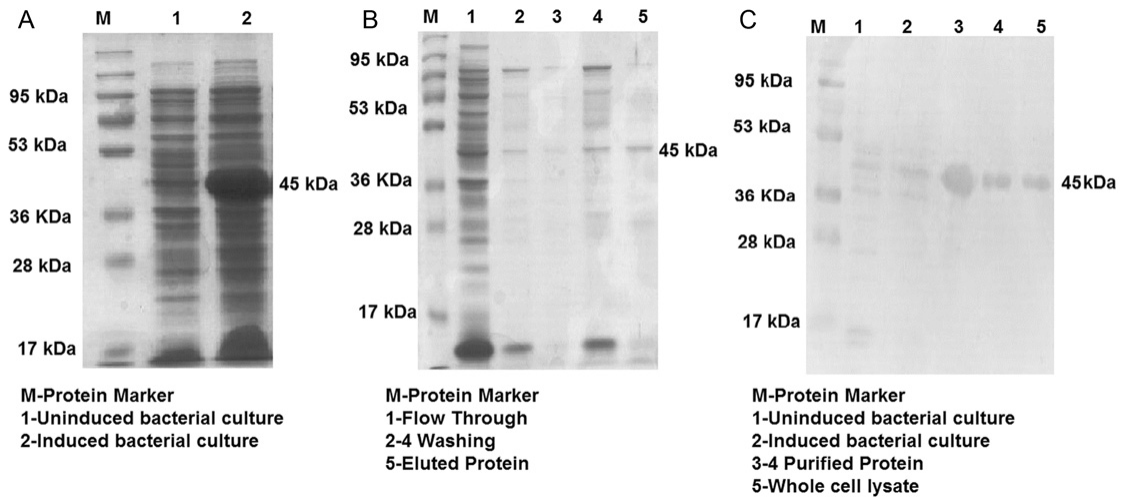
Expression of recombinant LdSir2RP in E. coli Rosetta strain and its purification. Expression and purification of rLdSir2RP (1A, IB) in prokaryotic cells, Whole Cell Lysate (WCL) of transformed *E*. *coli* separated on 12% acrylamide gel and stained with Coomassie blue, M: Molecular wt. markers; Lane 1: WCL before IPTG induction; lane 2: WCL after IPTG (1.0 mM) induction at 37°C. Lane 5: eluted protein (1B). Western blot analysis using anti-rLdSir2RPAb in uninduced WCL, induced WCL and SLD—M: Mol wt marker, Lane 1: uninduced WCL, Lane 2: induced WCL (1C).

The rLdSir2RP gene of *L*. *donovani* was successfully amplified (S1A Fig), T/A cloned (S1B Fig) and sequenced which was 99% homologous with *L*. *infantum* LiSir2RP. Further the protein share only ~35% identity with human counterpart thus is unlikely to trigger autoreactivity in humans. It was further sub-cloned in bacterial expression vector pET28a^+^ (S1C Fig) which revealed that the size of the expressed protein was ~45 kDa (Fig. 1A) and the rLdSir2RP was eluted at 250mM imidazole concentration (Fig. 1B). Immunoblots of lysates from *L*. *donovani* promastigote was performed with the polyclonal anti-rLdSir2RP antibody which detected one dominant protein of ~45 kDa (Fig. 1C). The presence of the lipopolysaccharides (LPS) content of the recombinant proteins was measured by Limulus amoebocyte lysate test (QCL-1000, Lonza, Walkersville, MD. USA) and was found to be below 10 endotoxin units (EU)/mg of the recombinant protein.

### Immunolocalization of rLdsir2RP in *Leishmania* parasite with Fluorescence microscopy

Immuno-localization study was carried out to study the sub-cellular location of rLdSir2RP enzyme, with its antibody raised in swiss mice. The protein was found to be localised in the cytoplasm of the cell (S2 Fig).

### Bioinformatical analysis

Phylogenetic un-rooted tree (S3a Fig) was generated of given sequences namely LinJ26V3 (*L*. *infantum*), LmjF26.021 (*L*. *major*), LbrM26V2 (*L*. *braziliensis*), Tb927.7.16 (*Trypanosoma brucei*), Sp|Q8 [XJB| (Human), Sp|O28597| (*Archaeoglobus fulgidus*) by the use of Clustal W2 software at EBI, MEGA5.2 tool. Interestingly, NAD sequence showed high similarity and low divergence with LinJ26V3 sequence and comes in the same clade-1. Similarly, LmjF26.021 comes in same clade-2 with clade-1. LbrM26V2 comes in same clade-3 with clade-2. Tb927.7.16 forms clade-4 with clade-3. Sp|Q8 [XJB| and Sp|O28597| showed less similarity with other sequences and form separate clade-5. The scale used here showed nucleotide substitution per site. Common motif was identified in all the sequences by the use of GLAM2 software of MEME suite having regular expression **FYSIA[KR]E[ML]?[KDN]?LWPGHFQPTAVHHFIRLLQD[KE] GRLLRCCTQNIDGLE[KR]AAGV.** By the use of CDART tool, curiously, it was found that Motif identified belongs to be the SIR2 superfamily of proteins (S3b Fig) which includes silent information regulator 2 (Sir2) enzymes which catalyze NAD+-dependent protein/histone deacetylation. Thus motif show deacetylase activity and since it is NAD+ dependent so it should have NAD binding pocket. Similar results were retrieved by Conserved Domain Database and SMART tool. Above results were further verified by the biochemical activity.

### T-Cell epitope prediction

Prediction of 9-mer and 15-mer promiscuous peptides for MHC-1 [47] and MHC-II [48] respectively was done by IEDB server using alleles which have been found to be the most common in North Indian sub population. Promiscuous epitopes having percentile score ≤1 were selected. BlastP search was performed to identify the identity with human protein if any and peptides showing high identity (>80) were not considered. Finally, for MHC-I alleles seven potential promiscuous epitopes were listed from LdSir2RP protein in (S1 Table) Similarly, for MHC-II alleles seven promiscuous peptides were listed in (S2 Table).

### Biochemical analysis of rLdSir2RP

Glutaraldehyde cross linking was employed to determine oligomeric state of native *L*. *donovani* SIR2 deacetylase. SDS PAGE analysis of cross linked protein with increasing concentration of glutaraldehyde has been shown in (S4 (1) Fig) in gel pic 2–4 position. A single band at the position near 45 kDA appeared with the increasing concentrations of glutaraldehyde. Similar was the situation in the absence of glutaraldehyde as evident in (S4 (1) (1) Fig) in 1 position. In both the cases, oligomeric state of the protein was not affected and it was observed as monomer. We further have analyzed NAD depended deacetylase activity of rLdSir2RP employing acetylated peptide as substrate for SIR2 deacetylase. A fluorophore and quencher were coupled to the amino and carboxyl terminal of substrate peptide. Separation of fluorophore and quencher by endopeptidase after deacetylation was resulted into the increase in fluorescence intensity. The intensity increased with the increase in substrate concentration as shown in (S4a Fig). Addition of NAD to the reaction mixture resulted into the increase in fluorescence intensity which was negligible in the absence of NAD (S4b Fig). Increase in protein concentration also increased the fluorescence intensity as shown in (S4c Fig).

### rLdSir2RP induced significant lymphoproliferative and NO responses in cured hamsters

**Fig 2:**
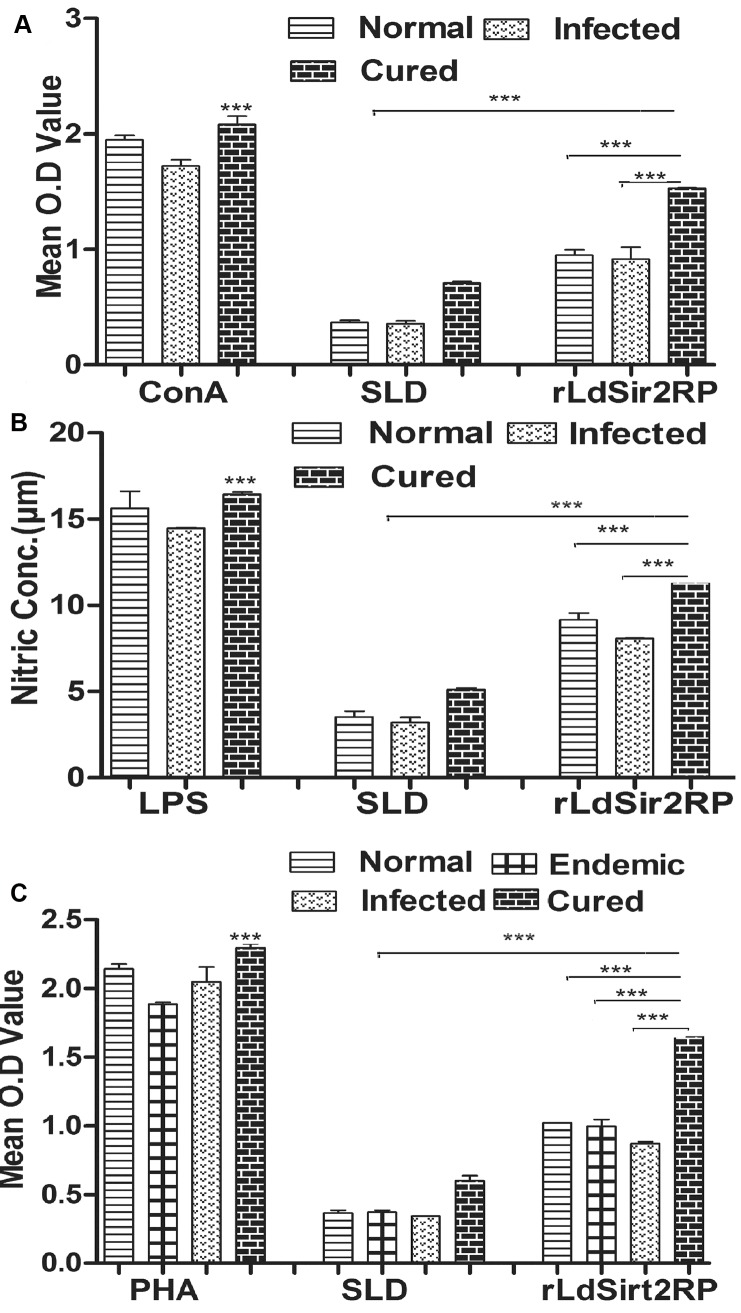
Cellular responses of rLdSir2RP of L.donovani in hamsters. (a) XTT response of mononuclear cells (lymph nodes) from normal, *L*. *donovani* infected (30 day p.c.) and treated hamsters in response to Con A, SLD and rLdSir2RP at 10 μg/ml each. Proliferation was represented as mean OD of stimulated culture—mean OD of unstimulated control. Each bar represents the pooled data (mean ± S.D. value) of 6 hamsters and the data represent the means of triplicate wells ± the S.D. Nitric oxide production (μM) by (b) J774A.1 cell line. The J774 A.1 cells were primed with the supernatants of stimulated lymphocytes (3 days with mitogen and 5 days with Ags) of normal, infected and cured hamsters in response to rLdSir2RP, SLD and LPS respectively at 10 μg/ml each. The estimation of NO production was done using Greiss reagent in supernatants collected from macrophage cultures 24 h after incubation and the absorbance of the reaction product was measured at 540 nm. Significance values indicate the difference between the SLD and rLdSir2RP (***, p< 0.001). **(c)** XTT response of PBMC from normal, *L*.*donovani* infected, and cured patients in response to PHA, SLD and rLdSir2RP at 10 μg/ml each. Proliferation was represented as mean OD of stimulated culture—mean OD of unstimulated control. Each bar represents the data (mean ± S.D. value) of triplicate wells. Significance values indicate the difference between the SLD and rLdSir2RP stimulation (***, p<0.001).

The cellular responses of lymph node cells of cured hamsters were assessed by XTT against the mitogen, i.e. Con A as well as SLD and rLdSir2RP. The responses were compared with that of normal as well as *L*. *donovani* infected groups that served as controls. The normal control as well as cured *Leishmania* infected group had shown significantly higher proliferative responses against Con A as compared to *L*. *donovani*-infected group (Fig. 2a). The proliferative response of PBMCs against rLdSir2RP was significantly higher (2 folds) in cured/infected hamsters. The difference was statistically significant (***, p< 0.001).

NO-mediated macrophage effector mechanism is known to be critical in the control of parasite replication in the animal model hence its production in macrophage cell line J774 A.1, was studied after 24 h of incubation in the presence of rLdSir2RP and SLD. For comparison, NO production in mitogenic (LPS) stimulated and unstimulated cells served as positive and negative controls respectively (Fig. 2b). NO production was recorded to be higher against rLdSir2RP (***, p< 0.001).

### rLdSir2RP stimulates PBMCs from cured *Leishmania* patients to proliferate and to express a predominant Thl cytokine profile

We further validated the cellular responses (XTT and cytokine levels) of rLdSir2RP in PBMCs of cured patients, endemic and non-endemic controls and *L*. *donovani* infected donors. Individual donors in each study group were found to elicit different responses. Lymphoproliferation and cytokine responses of PBMCs from patients with active VL/cured/endemic were compared using rLdSir2RP and SLD. Endemic control and cured patients exhibited relatively higher mean OD values against PHA respectively compared to unstimulated control. PBMCs from all the cured and active VL patients proliferated in response to rLdSir2RP with mean OD values which was higher than SLD mean OD values. Values were statistically significant (***, p<0.001) (Fig. 2c). The results demonstrate that rLdSir2RP is a potent T-cell antigen recognized by a majority of *L*. *donovani* infected/cured/endemic individuals in different stages or manifestations of infection.

**Fig 3:**
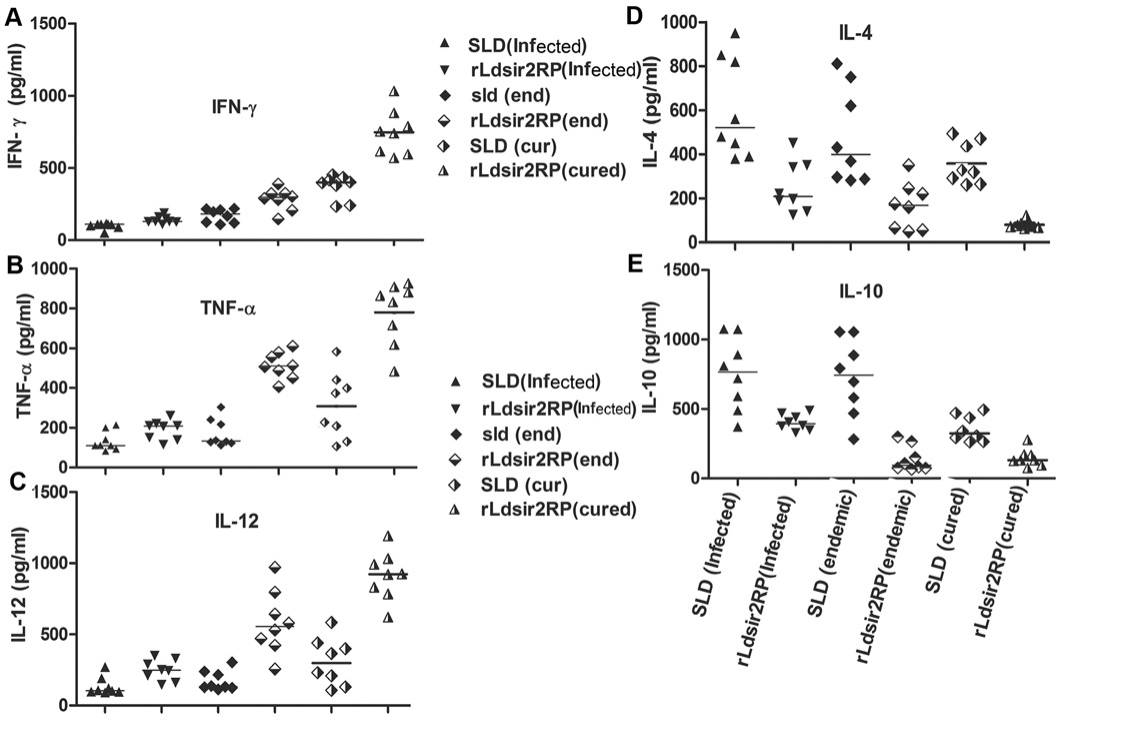
Th1 and Th2 cytokine production. Th1 cytokine (a) IFN-γ, (b) TNF-α, (c) IL-12 and Th2 cytokines (d) IL-4, (e) IL-10 production in PBMCs from individuals of cured VL patients (8), infected individuals (8) and endemic controls (8) in response to rLdSir2RP and SLD Ags, each data point represents one individual. The X axis refers to groups of individuals (CUR, infected and END) and the Y-axis corresponds to the values of respective cytokine as concentrations in pg/ml. The levels of IFN- (a) was observed to be 2.5 to 3.5 fold higher in the supernatants of cured patients respectively as compared to endemic contacts and infected patients. Low levels of IL-4 (d), IL-10 (e) cytokines against rLdSir2RP were detected in supernatants of cured patients followed by endemic contacts and infected patients. The mean concentration of cytokine for each group is indicated by the horizontal bars. Values are given as concentration in pg/ml.

To assess the Th1/Th2 stimulatory potential of the rLdSir2RP we further studied the cytokine levels viz. IFN-γ, IL-12 as well as IL-10 in PBMCs from cured/infected patients as well as in endemic contacts against rLdSir2RP. The levels of IFN- was observed to be 2.5 to 3.5 fold higher in the supernatants of cured patients respectively as compared to endemic contacts and infected patients. IFN- is very important protective type of cytokines which is very high in cured patients. On the contrary, very low level of IL-10 and IL-4 cytokines against rLdSir2RP was detected in supernatants of cured patients followed by endemic contacts and infected patients. PBMCs of cured/ endemic contacts generated a mixed ThI/Th2 cytokine profile against SLD wherein high levels of IL-10/IL-4 and very little level of IFN-, and IL-12 were noticed in response to SLD in infected patients. The ratio of the Th1/Th2 (IFN-/IL-10) cytokines in cured patients was 4.0 against the rLdSir2RP and 1.15 against the SLD. (Fig. 3).

### rLdSir2RP modulates *Leishmania*-specific IgG and its isotypes in naïve hamsters as well as in cured and endemic patient sample

**Fig 4:**
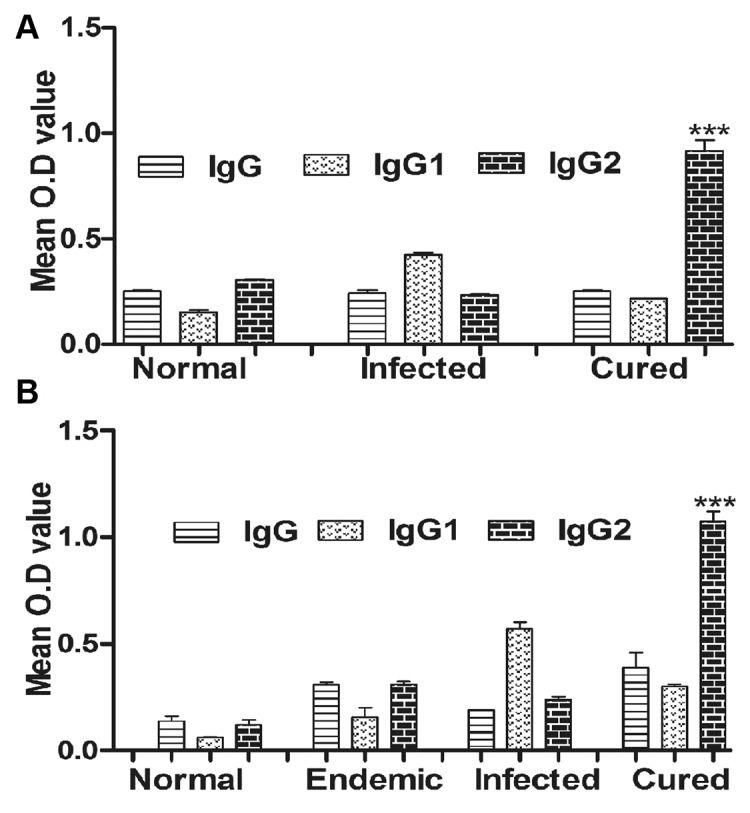
Determination of antibody response in hamsters/patients. (Fig 4a) Ab levels (OD value) in hamster’s serum with rLdSir2RP. Each bar represents the pooled data (mean±S.D. value) of three replicates. Significance values indicate the difference between the cured group and normal group (***, p<0.001). (Fig 4b). Ab levels (OD value) in patient sample with rLdSir2RP. Each bar represents the pooled data (mean±S.D. value) of three replicates. Significance values indicate the difference between the endemic group and cured group (***, p<0.001).

To assess the antibody level in the serum of cured animals, we further estimated the level of IgG and its isotypes in response to rLdSir2RP. In general, we observed significantly higher (~2 to 3 fold) IgG2 response in cured animals as compared to the normal and infected animals (Fig. 4a) against rLdSir2RP, but there was no apparent difference in the IgG1 response between the cured and the normal animals against any of them.

We further validated/estimated the level of IgG and its isotypes in cured/infected *Leishmania* patients. We observed significantly higher (~2 to 3 fold) IgG2 response by cured patients as compared to the endemic contacts and infected patients. However, there was no apparent difference in the IgG1 response between the endemic and the cured patients (Fig. 4b). Significance values indicate the difference between the endemic group and cured group (***, p<0.001).

### Immunization with recombinant rLdSir2RP induced optimum prophylactic efficacy against *L*. *donovani* challenges

The hamsters immunized with rLdSir2RP alongwith BCG, in general, gained considerable weight as compared to those groups of hamsters which were immunized with BCG alone as well as to unimmunized infected animals, when kept simultaneously upto day 90 post challenge (p.c).

**Fig 5:**
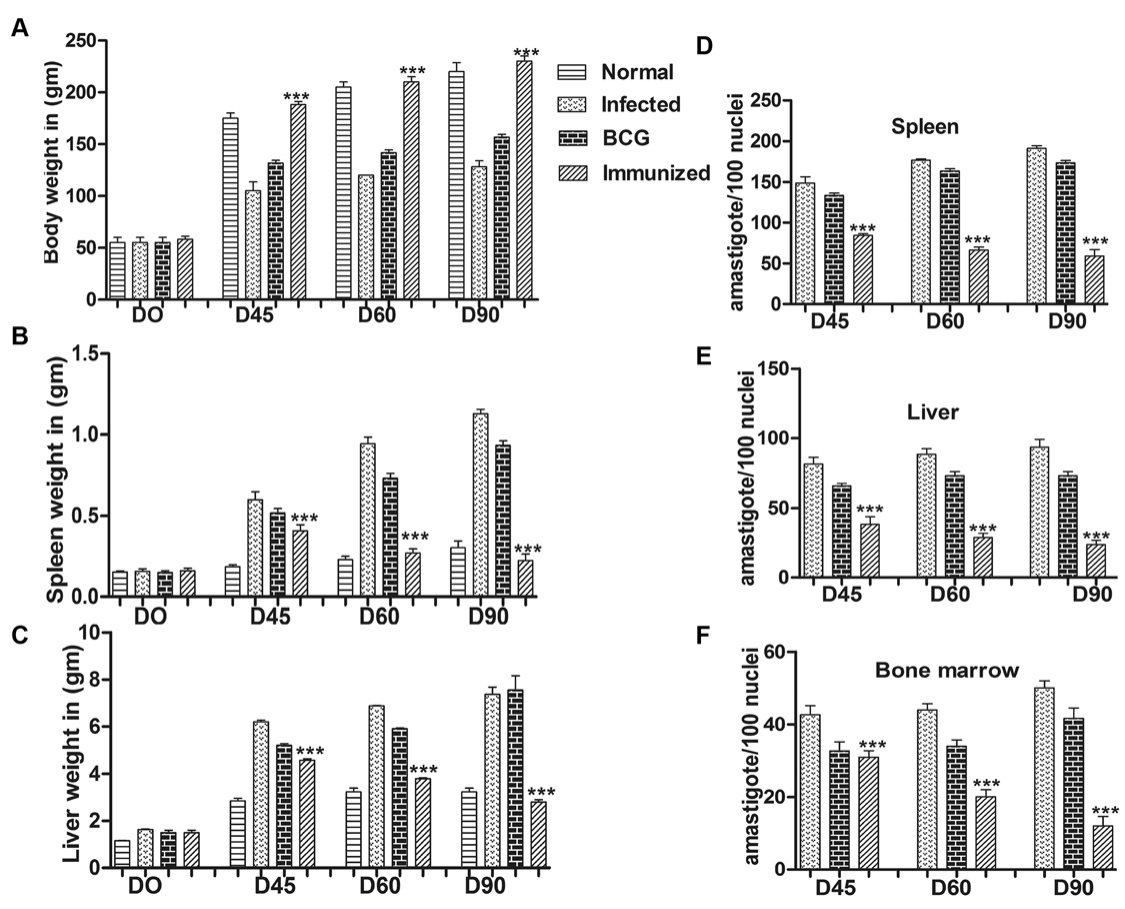
Parasite burden. Clinical outcomes following *L*. *donovani* challenge in hamsters immunized with rLdSir2RP+BCG. On day 21 after the booster, the hamsters of infected, BCG alone and vaccinated groups were challenged intracardially with 10^**7**^ metacyclic promastigotes of *L*. *donovani*. Following parameters observed Body weight (a), spleen weight (b), liver weight (c), Parasite burden (no. of amastigotes per 100 cell nuclei) in the spleen(d), liver(e), and bone marrow (f) on days 45 and 60 p.c. Significance values indicate the difference between the vaccinated groups and infected group (***, p<0.001). Data represent mean values standard errors (SE) at the designated time points.

The hamsters immunized with rLdSir2RP+BCG and challenged with *L*. *donovani* exhibited an optimal reduction in parasite load (~75%) in spleen, liver and bone marrow on day 90 p.c which was significantly higher (***, p<0.001) than all the other experimental as well control groups. The survival of rLdSir2RP+BCG immunized hamsters after the *Leishmania* challenge was the longest (6 months post-infection) as compared to the unimmunized ones (day 60 to 90 p.c.) wherein progressive increase in parasite load was observed (Fig. 5).

**Fig 6:**
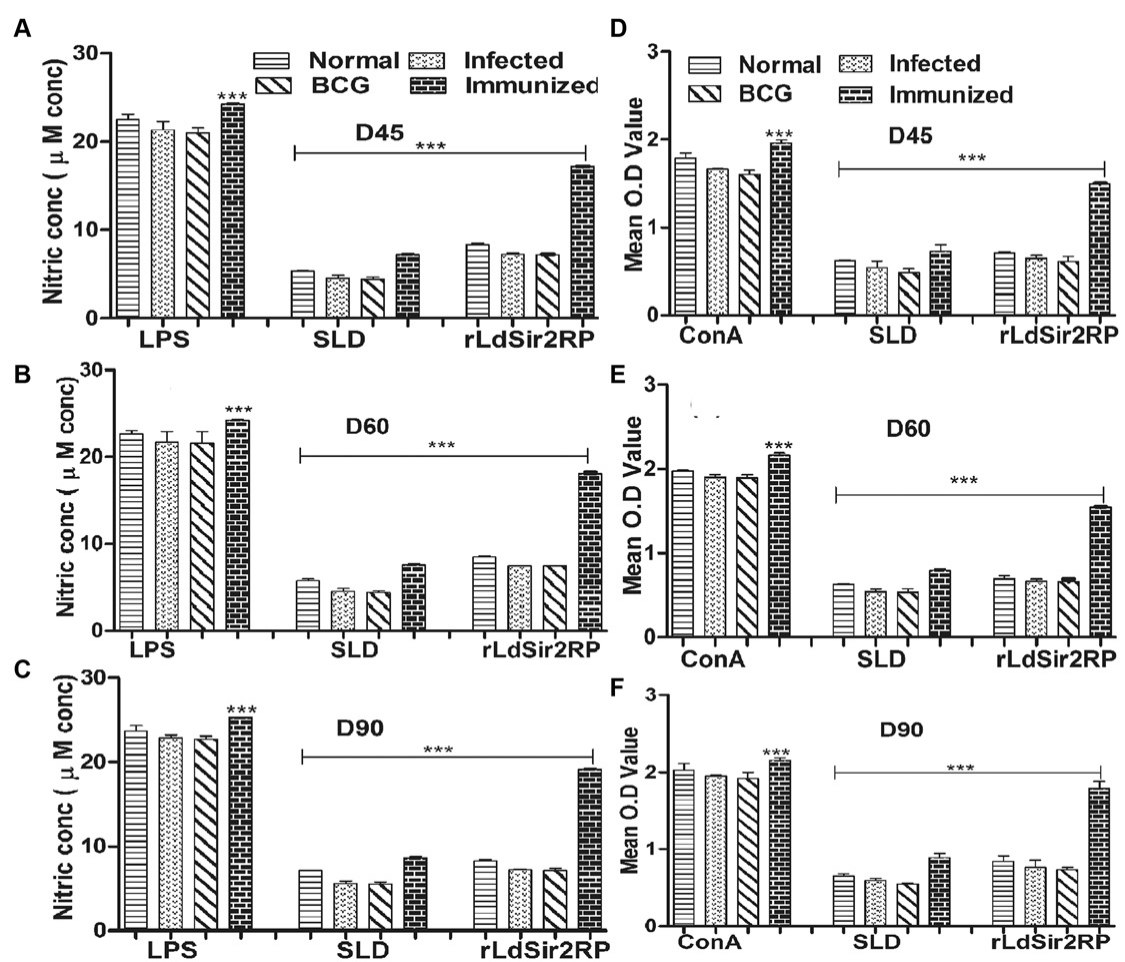
Cellular responses of rLdSir2RP of L.donovani in vaccinated hamsters. Nitric oxide production (μM) (a, b, c) by J774A.1 cell line. The J774 A.1 cells were primed with the supernatants of stimulated lymphocytes (3 days with mitogen and 5 days with Ags) of normal, infected and vaccinated hamsters in response to rLdSir2RP, SLD and LPS respectively at 10 μg/ml each. The estimation of NO production was done using Greiss reagent in supernatants collected from macrophage cultures 24 h after incubation and the absorbance of the reaction product was measured at 540 nm. XTT response (d, e, f) of mononuclear cells (lymph nodes) from normal, *L*. *donovani* infected and vaccinated hamsters in response to Con A, SLD and rLdSir2RP at 10 μg/ml each Proliferation was represented as mean OD. The data represent the means of triplicate wells ± S.D. Significance values indicate the difference between the SLD and rLdSir2RP stimulation (**, p < 0.01; ***, p<0.001).

NO production and lymphoproliferative response was also recorded to be significantly higher in hamsters immunized with rLdSir2RP group wherein an optimum stimulation to the tune of 2.5 to 3.0 folds was observed in comparison to unvaccinated infected control group of hamsters and this was highly significant (Fig. 6). The cell proliferation and nitrite production in the hamsters receiving SLD was also significantly higher (~2.0–2.5 fold). Significance values indicate the difference between the SLD and rLdSir2RP stimulation (**, p < 0.01; ***, p< 0.001). However, BCG vaccinated group exhibited parasite load and immune responses similar to infected control (Figs. 5, 6).

### Immunization with rLdSir2RP generates Th1-type cytokine profile as determined by qRT-PCR and IgG2 type antibody response

**Fig 7:**
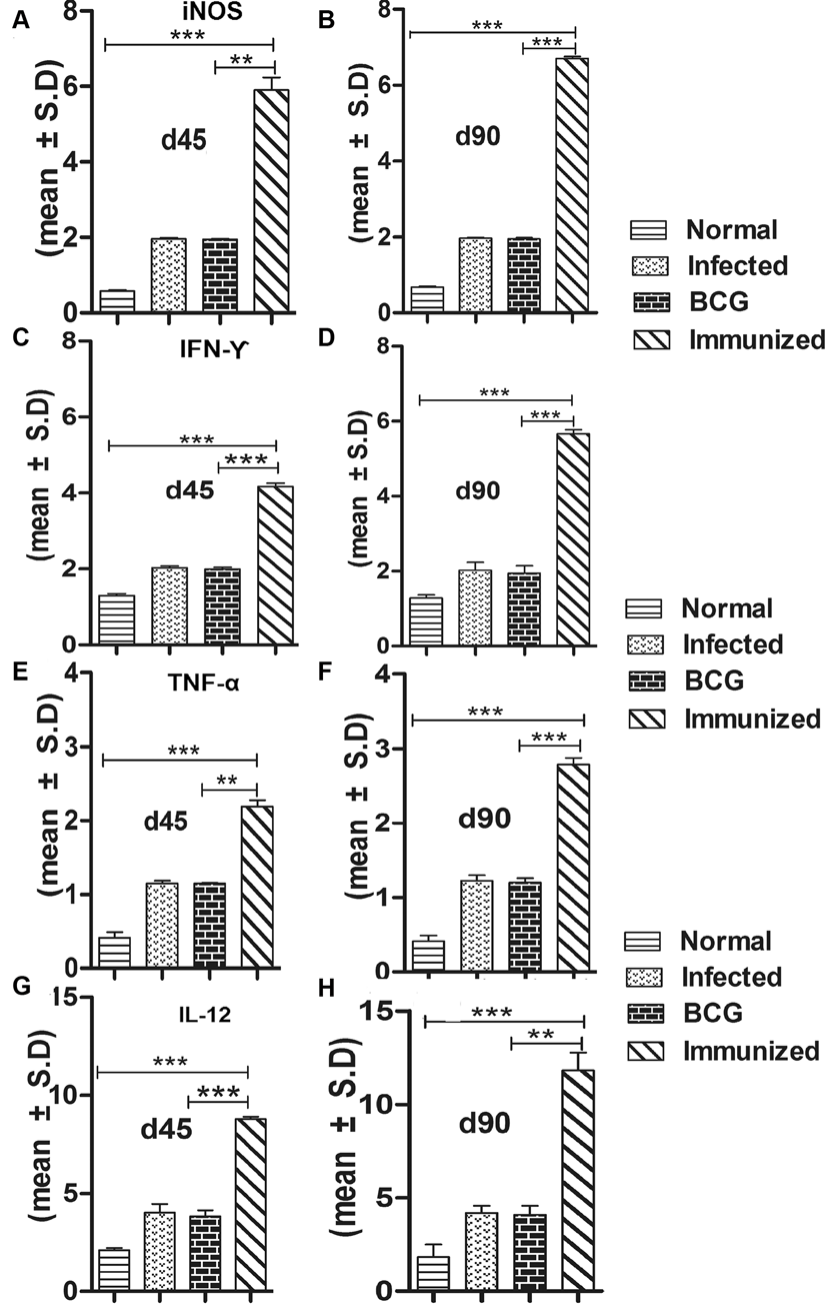
Splenic iNOS and Th1cytokine mRNA expression. Splenic iNOS and cytokine mRNA relative fold expression profile analysis of normal and immunized hamsters on days 45 p.c. and 90 p.c by quantitative real-time RT-PCR. The expression of Th1 cytokines viz. iNOS(a,b), IFN-γ (c,d), TNF-α (e,f) as well as IL-12 (g,h) was also observed to be ~2.10 folds higher (p<0.001) in the hamsters of rLdSir2RP vaccinated group as compared to the other experimental as well as control groups. Significance values indicate the difference between the vaccinated group and infected group (**, p<0.01; ***, p<0.001).

**Fig 8:**
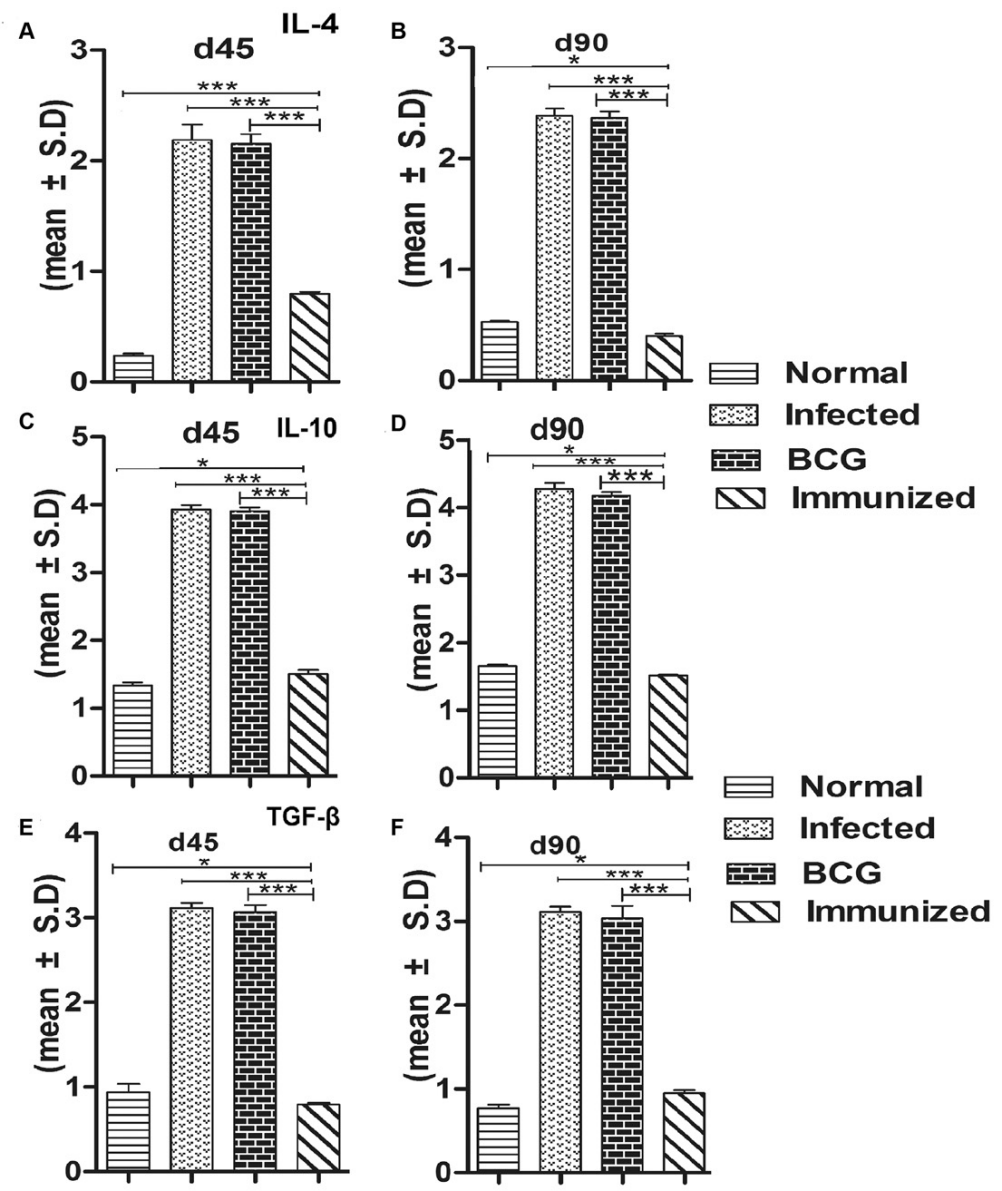
Th2 cytokine mRNA expression. Th2 cytokines mRNA expression profile analysis of normal and immunized hamsters on days 45 p.c.and 90 p.c were checked by quantitative real-time RT-PCR. The expression of Th2 cytokines viz. IL4 (a,b), IL10 (c,d), TGFß (e,f) was also observed to be ~3.00 folds higher (p<0.001) in the Infected hamsters as compare to vaccinated group as well as control groups. Significance values indicate the difference between the vaccinated group and infected group (***, p<0.001), vaccinated group and BCG group (***, p<0.001), vaccinated group and normal group (*, p<0.05).

**Fig 9:**
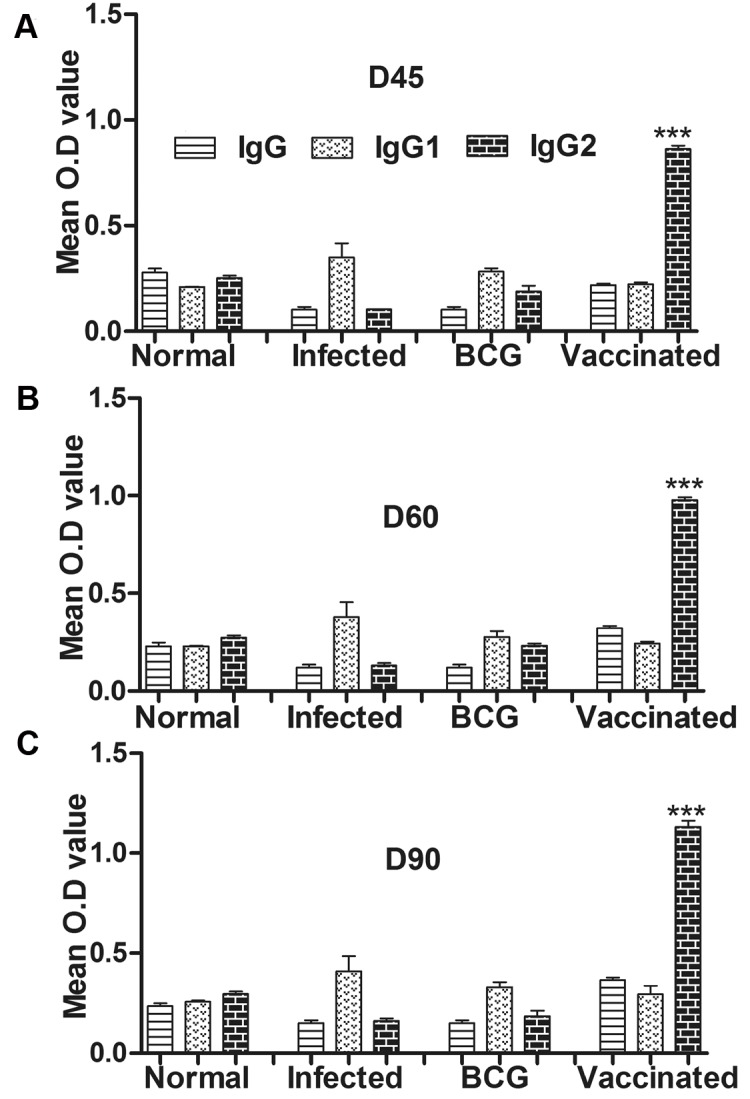
Leishmania-specific IgG and its isotypes IgG1 and IgG2 responses. Serum samples were collected from different groups of hamsters at designated time points and assayed for rLdSir2RP—specific IgG, IgG1, and IgG2 levels by ELISA. Antibody responses in rLdSir2RP vaccinated hamsters in comparison to the unimmunized infected hamsters on days 45, 60 and 90 p.c. The IgG1 were observed to be elevated by 1 to 2 fold in infected control group in comparison to the group of immunized hamsters with rLdSir2RP at the time interval of Days 45(a), d60(b) and d90(c). There was significant elevation of IgG2 level (by 3.5 to 4 folds) in group of hamsters immunized with rLdSir2RP at the time interval of Days 45(a), d60(b)and d90(c). Data are presented as the absorbance at 492 nm and are means ± SE for 3–4 hamsters per group in triplicate wells. Significance values indicate the difference between the vaccinated group and infected group (***, p<0.001).

For further assessment of cellular immune response in hamsters immunized with rLdSir2RP the expression of Th1 and Th2 mRNA cytokines was measured by qRT-PCR. The iNOS transcript was found to be optimally and significantly up-regulated by ~3.0 fold (p<0.001) in hamsters immunized with rLdSir2RP as compared to that of *L*. *donovani* infected group. The expression of Th1 cytokines viz. TNF-α as well as IFN-γ was also observed to be ~2.10 fold higher (p<0.001) in the hamsters of rLdSir2RP vaccinated group as compared to the control groups (Fig. 7). Similar was the case with IL-12 expression which was also significantly (p< 0.001) increased by ~2.0 fold in rLdSir2RP immunized hamsters. On the other hand, the expression of Th2 cytokines particularly IL-10, IL-4 and TGF-β, known to be associated with progressive VL, were significantly down-regulated by ~3.0 to ~3.5 fold in rLdSir2RP vaccinated hamsters (p<0.001) (Fig. 8). Significance values indicate the difference between the NAD vaccinated group and infected group (***, p< 0.001), infected group and the normal group (**, p < 0.01), vaccinated group and normal group (*, p < 0.05), vaccinated group and BCG group (***, p < 0.001). The immune response was similar in BCG vaccinated group and infected control (Figs. 7, 8). The IgG1 were observed to be elevated by 1 to 2 fold in infected control group in comparison to the group of immunized hamsters with rLdSir2RP at the time interval of days 45, 60 and 90 p.c. On the other hand there was significant elevation of IgG2 level (by 3.5 to 4 folds) in group of hamsters immunized with rLdSir2RP (Fig. 9). As a measure of CMI, the elevation of IgG2 was indicator of the development of effective immune responses. Significance values indicate the difference between the NAD vaccinated group and infected group (***, p<0.001). The antibody responses generated in BCG vaccinated group was at par to infected control (Fig. 9).

## Discussion

Silent information regulator 2 gene (Sir2), identified through immunoproteomics as one of the potential Th1 stimulatory proteins in the sub-fraction of soluble lysate of *L*. *donovani* ranging from 89.9 to 97.1 kDa [11,49]. It was first discovered in *Saccharomyces cerevisiae* and was named after its ability to relieve gene silencing [50]. Once discovered, sirtuins were rapidly characterized in yeast, bacteria, plants and mammals. Sirtuins have been involved in metabolic and chromatin regulation regulates ribosomal DNA recombination, gene silencing, DNA repair, chromosomal stability and longevity throughout evolution, the first examples of chromatin-like organization of DNA in archaea [51, 52]. Sirtuin family is very large having various types of Sir1, Sir2, Sir3, Sir4, Sir5, Sir6, and Sir7 in human. NAD-dependent SIRT2 protein and its orthologs have also shown as crucial regulators for aging and longevity in various organisms such as yeast, worms, and flies [53–57]. SIRT2 was also involved in cellular processes such as neuroprotection and the inflammatory response [58]. This protein is also being the good candidate targets for anticancer drugs and therapies [59]. In *Leishmania* parasites this protein is reported to be involved in essential role in their survival and virulence [60]. This has led to the generation of SIRT2 knock out mutant cell lines and was evaluated for their prophylactic potential. This protein has been attributed to have essential role in survival and induces B-cell differentiation and *in vivo* production of specific antibodies thus can serve as a good vaccine target [60]. Herein, we have explored the immunogenicity and prophylactic potential of recombinant SIRT2 protein of *L*. *donovani*, the causative organism of VL prevalent in Indian subcontinent. For the purpose, the Sir2 gene of *L*. *donovani* was cloned, expressed and purified and subjected to biochemical and bioinformatics characterization for verification. The LdSir2RP gene belonging to 1122 bp was successfully cloned which has 98%, 95% and 45% homology with *L*. *infantum*, *L*. *major* and human respectively. Immunoblot study of *L*. *donovani* promastigote lysates with the polyclonal anti-LdSir2RP antibody raised in mouse revealed one dominant protein of ~45 kDa. This protein was identified earlier at higher molecular weight range in proteomic studies which is in contrast to its observed molecular mass. This could be attributed to the post-translational modifications which are widely prevalent in *Leishmania* [49]. Bioinformatics analyses have further shown that LdSir2RP have NAD binding domain and deacetylase motif also and activity was confirmed by fluromatric assay. Unlike yeast, the recombinant protein has cytoplasmic localization as also observed in other *Leishmania* sp [14]. It exists in monomeric form as was further proven by crosslinking experiment using glutaraldehyde.

In our earlier studies we have demonstrated that a T-cell response develops when cells from cured individuals are stimulated with SLD and its subfractions [11,49]. The ability of rLdSir2RP, derived from soluble *Leishmania* antigen to induce cellular immune response was re-assessed in *Leishmania* infected and cured hamsters as the systemic infection of the hamster with *L*. *donovani* is very similar to human Kala-azar as it results in a relentlessly increasing visceral parasite burden, progressive cachexia, hepatosplenomegaly, pancytopenia, hypergammaglobulinemia, and ultimately, death [46]. Then its immunogenicity was further validated in endemic non-immune donors (household contacts without any clinical symptoms) and in immune patients of VL that were cured either with Amphotericin B and/or Miltefosine. As observed earlier the cellular responses to rLdSir2RP were similar in endemic controls and in cured patients of VL as well as in hamsters indicating that the results so obtained with the hamster could be translated into humans [61].

It is well established that recovery from *Leishmania* infection [62,63] with production of IFN- and IL-12 and enhanced expression of iNOS [64]. In this study we have observed significantly higher cellular responses viz. lymphoproliferative as well as NO release against all the immunised hamsters in comparison to normal and infected ones. In case of human subjects, the presence of a positive immune response in all the eight endemic contacts suggests that the frequency of subclinical infection in an endemic area such as Bihar is high, as has been reported in other areas of the world [65–68]. Further, it has been observed that whereas SLD stimulated PBMCs from *L*. *donovani* infected individuals elicit a mixed Thl and Th2 like immune response; rLdSir2RP shifted this pattern towards an exclusive Thl (IFN- and IL-12) cytokine profile. In addition, rLdSir2RP stimulated PBMCs from cured patient to produce IL-12, TNF-α and IFN- in contrast to SLD and elicited very low level cytokines in endemic contacts. Thus, these observations suggest that rLdSir2RP appears to have distinctive immunological properties and the responses may be associated with protective immunity.

We further assessed the prophylactic potential of rLdSir2RP in combination with BCG a well known adjuvant and observed that an optimal reduction in parasite load (~75%) in spleen, liver and bone marrow was observed on day 90 p.c which was significantly higher than all the control groups. The iNOS transcript as well as the expression of Th1 cytokines—TNF-α, IFN-γ and IL-12 was found to be optimally and significantly unregulated by ~2.0 to 3.0 fold in hamsters immunized with rLdSir2RP as compared to that of *L*. *donovani* infected group. On the other hand, the expression of Th2 cytokines particularly IL-10, IL-4 and TGF-β, known to be associated with progressive VL, were significantly down-regulated by ~3.0 to ~3.5 fold in rLdSir2RP vaccinated hamsters. Finally, unlike mice where IL-4 and IL-12 direct IgG subclass switching of IgG1 and IgG2a, respectively, such distinct IgG classes remains obscure in hamsters [69, 70]. These data, well supported by the elevation of IgG2, was indicator of the development of effective immune responses. Further, to further understand the molecular basis of the immune response elicited by the protein, epitope prediction for both MHC-I and MHC-II potential promiscuous epitopes from Ldsir2 sequence was carried out. Based on 9 to 15-mer long peptides LdSir2 had 7 epitopes each for MHC class I & II.

Thus, the ability of rLdSir2RP to protect hamsters considerably to *L*. *donovani* challenges generating a T-cell response, together with *in-silico* analysis suggests that if combined with other potential Th1 stimulatory proteins/peptides it may provide absolute prophylactic/therapeutic and life-long protection against VL.

## Supporting Information

S1 Fig rLdSir2RP was cloned and subcloned in expression vector. The rLdSir2RP gene of *L*. *donovani* was successfully amplified (S1A Fig), T/A cloned (S1B Fig). It was further sub-cloned in bacterial expression vector pET28a^+^ (S1C Fig).
(TIF)

S2 Fig Immunofluorescence analysis of the cytoplasmic distribution of rLdSir2RP in L. donovani promastigotes. (b) the images showing a diffusely pattern of rLdSir2RP throughout the cell body with a marked exclusion of the nucleus. a) nuclei and kinetoplasts labelled with DAPI; b) immuno-fluorescence images; c) differential interference contrast image; d) merged images.
(TIF)

S3 Fig Phylogenetic un-rooted tree. (a) Phylogenetic tree was generated of given sequences namely LinJ26V3 (*Leishmania infantum*), LmjF26.021 (*Leishmania major*), LbrM26V2 (*Leishmania braziliensis*), Tb927.7.16 (*Trypanosoma brucei*), Sp|Q8 [XJB| (Human), Sp|O28597| (*Archaeoglobus fulgidus*) by the use of Clustal W2 software at EBI, MEGA5.2 tool. (b) Common motif was identified in all sequences by the use of GLAM2 software of MEME suite having has regular expression FYSIA[KR]E[ML]?[KDN]?LWPGHFQPTAVHHFIRLLQD[KE]GRLLRCCTQNIDGLE[KR]AAGV By the use CDART tool, curiously it was found that Motif identified belongs to be the SIR2 super family of proteins.
(TIF)

S4 Fig Cross linking experiment. (1) SDS PAGE analysis of glutaraldehyde cross linked recombinant protein. M represents molecular marker. Lane1, 2, 3 and 4 represent 0, 0.5, 1 and 1.5% of glutaraldehyde. We observed single band at the position near 45 kDA with increasing concentration of glutaraldehyde. Similarly a single band was observed in the absence of glutaraldehyde as shown. (2) We have analyzed NAD depended deacetylase activity of recombinant SIR2 deacetylase. (S4a Fig) Represents substrate depended activity of recombinant protein, bar 0, 1 and 2 show 0, 1 and 2 micro molar concentration of substrate peptide in standard reaction. (S4b Fig) represents NAD depended activity. c. represents effect of increasing concentration on enzyme activity. (S4c Fig) Figures show the fluorescence measurement of enzyme reaction mixture.
(TIF)

S1 Table Indicating predicted MHC-I epitope sequences, their length, position and method used for identification. (DOC)

S2 Table Indicating predicted MHC-II epitope sequences, their length, position and method used for identification. (DOC)
