# Correction: Recombinant NAD-dependent SIR-2 Protein of *Leishmania donovani*: Immunobiochemical Characterization as a Potential Vaccine against Visceral Leishmaniasis

**DOI:** 10.1371/journal.pntd.0003742

**Published:** 2015-04-22

**Authors:** Rajendra K Baharia, Rati Tandon, Tanuj Sharma, Manish K Suthar, Sanchita Das, Mohammad Imran Siddiqi, Jitendra Kumar Saxena, Shyam Sundar, Anuradha Dube

The eighth author’s name is spelled incorrectly. The correct spelling is: Shaym Sundar.
