# Supplementary figures and images for: Recombinant NAD-dependent SIR-2 Protein of Leishmania donovani: Immunobiochemical Characterization as a Potential Vaccine against Visceral Leishmaniasis
Source: PLoS Negl Trop Dis. 2015 Mar 6;9(3):e0003557. doi: 10.1371/journal.pntd.0003557 (PMC4351947; doi:10.1371/journal.pntd.0003557)

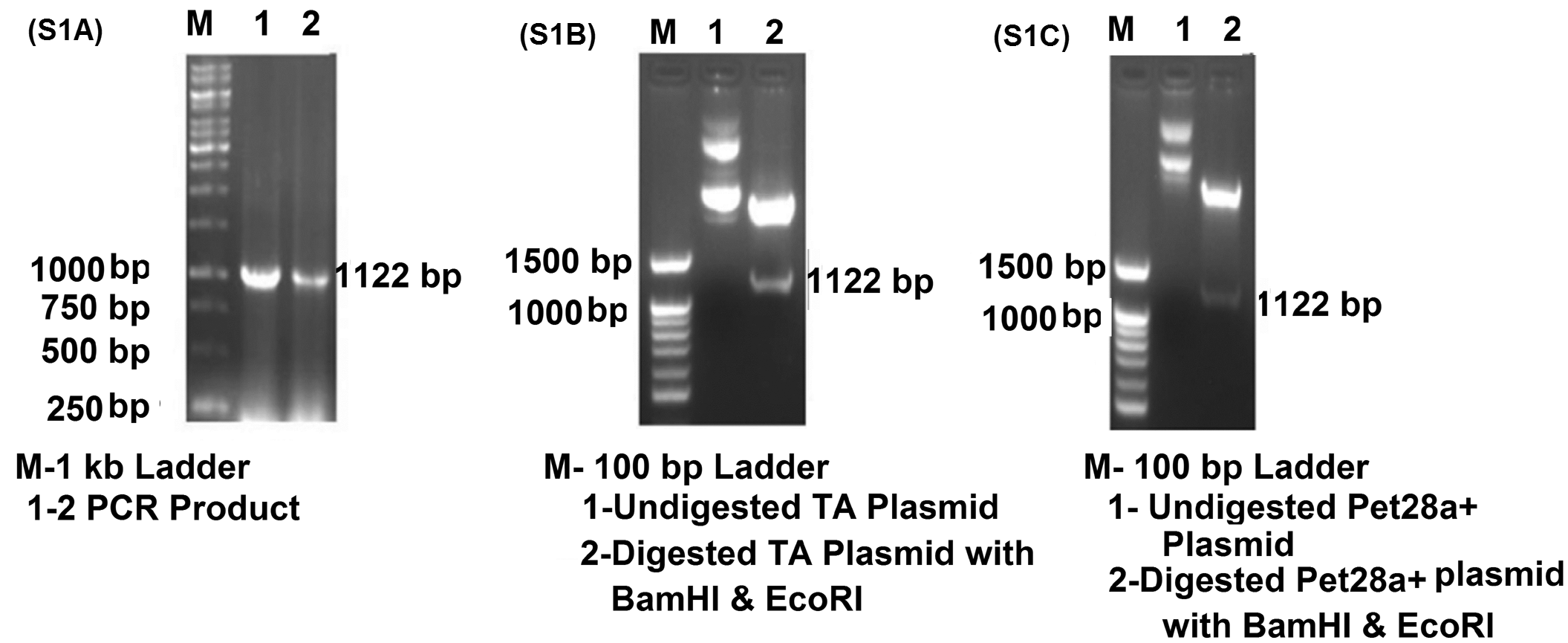

Supplement: S1 Fig — The rLdSir2RP gene of L. donovani was successfully amplified (S1A Fig), T/A cloned (S1B Fig). It was further sub-cloned in bacterial expression vector pET28a+ (S1C Fig). (TIF) [file pntd.0003557.s001.tif]

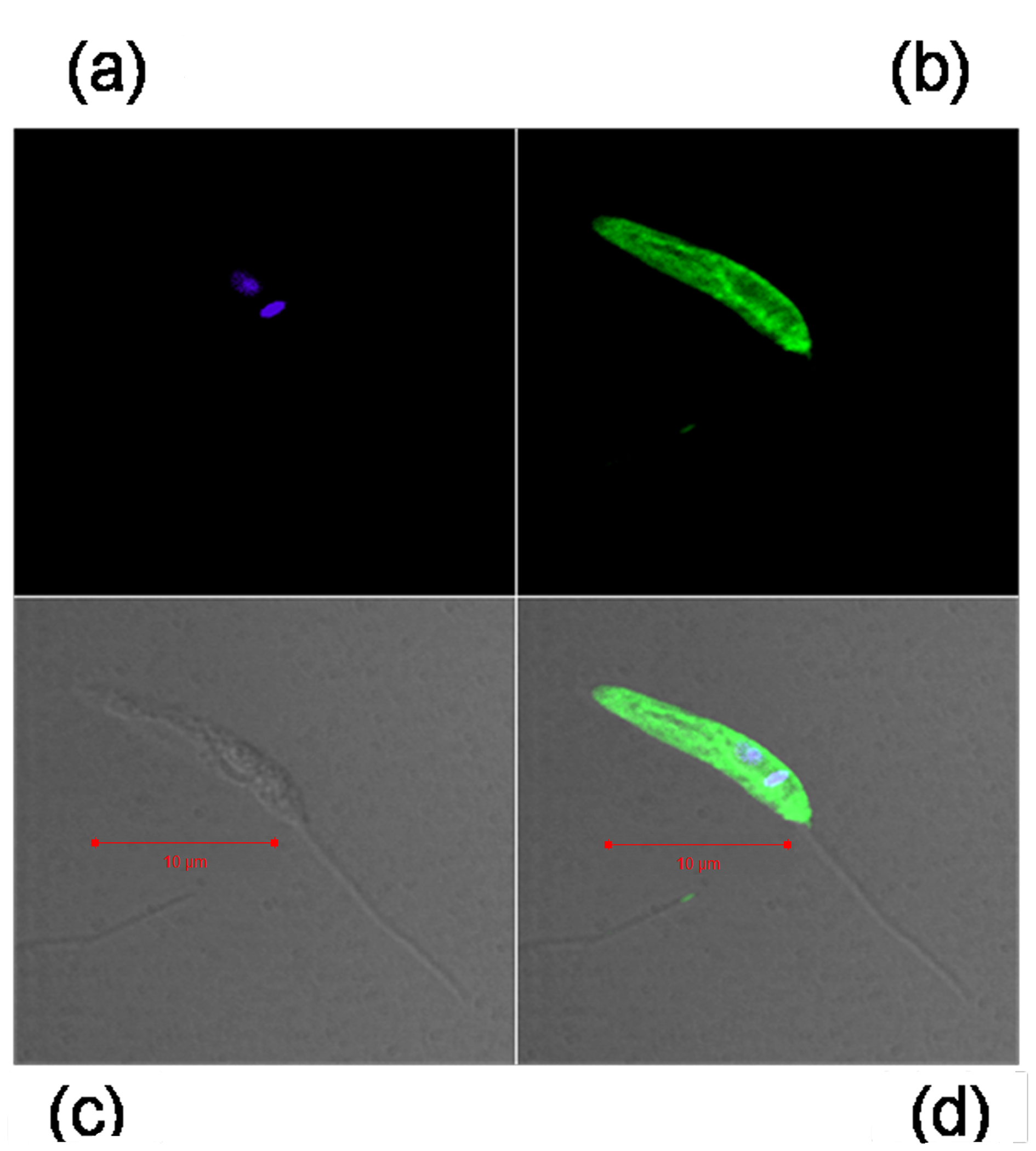

Supplement: S2 Fig — (b) the images showing a diffusely pattern of rLdSir2RP throughout the cell body with a marked exclusion of the nucleus. a) nuclei and kinetoplasts labelled with DAPI; b) immuno-fluorescence images; c) differential interference contrast image; d) merged images. (TIF) [file pntd.0003557.s002.tif]

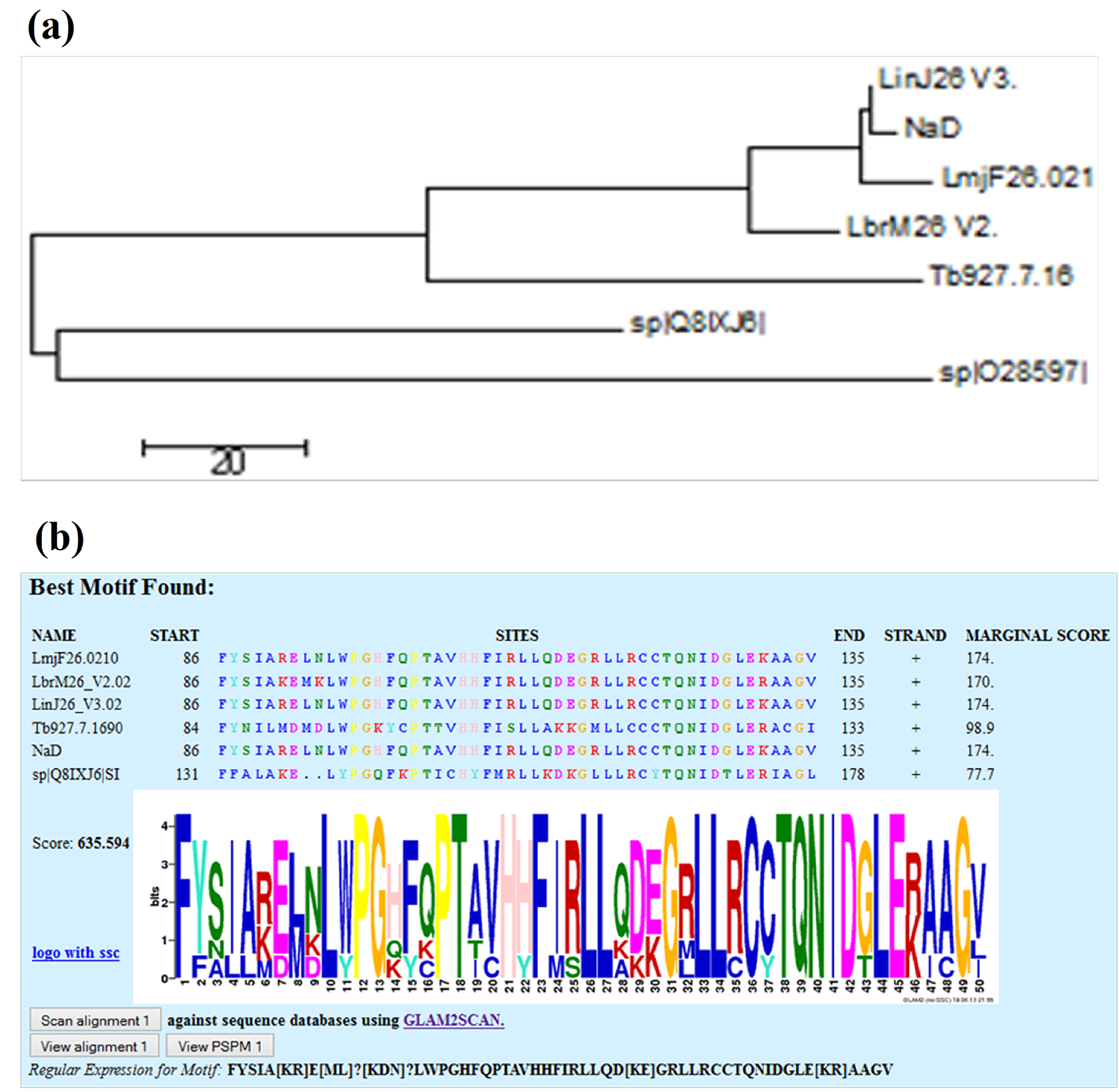

Supplement: S3 Fig — (a) Phylogenetic tree was generated of given sequences namely LinJ26V3 (Leishmania infantum), LmjF26.021 (Leishmania major), LbrM26V2 (Leishmania braziliensis), Tb927.7.16 (Trypanosoma brucei), Sp|Q8 [XJB| (Human), Sp|O28597| (Archaeoglobus fulgidus) by the use of Clustal W2 software at EBI, MEGA5.2 tool. (b) Common motif was identified in all sequences by the use of GLAM2 software of MEME suite having has regular expression FYSIA[KR]E[ML]?[KDN]?LWPGHFQPTAVHHFIRLLQD[KE]GRLLRCCTQNIDGLE[KR]AAGV By the use CDART tool, curiously it was found that Motif identified belongs to be the SIR2 super family of proteins. (TIF) [file pntd.0003557.s003.tif]

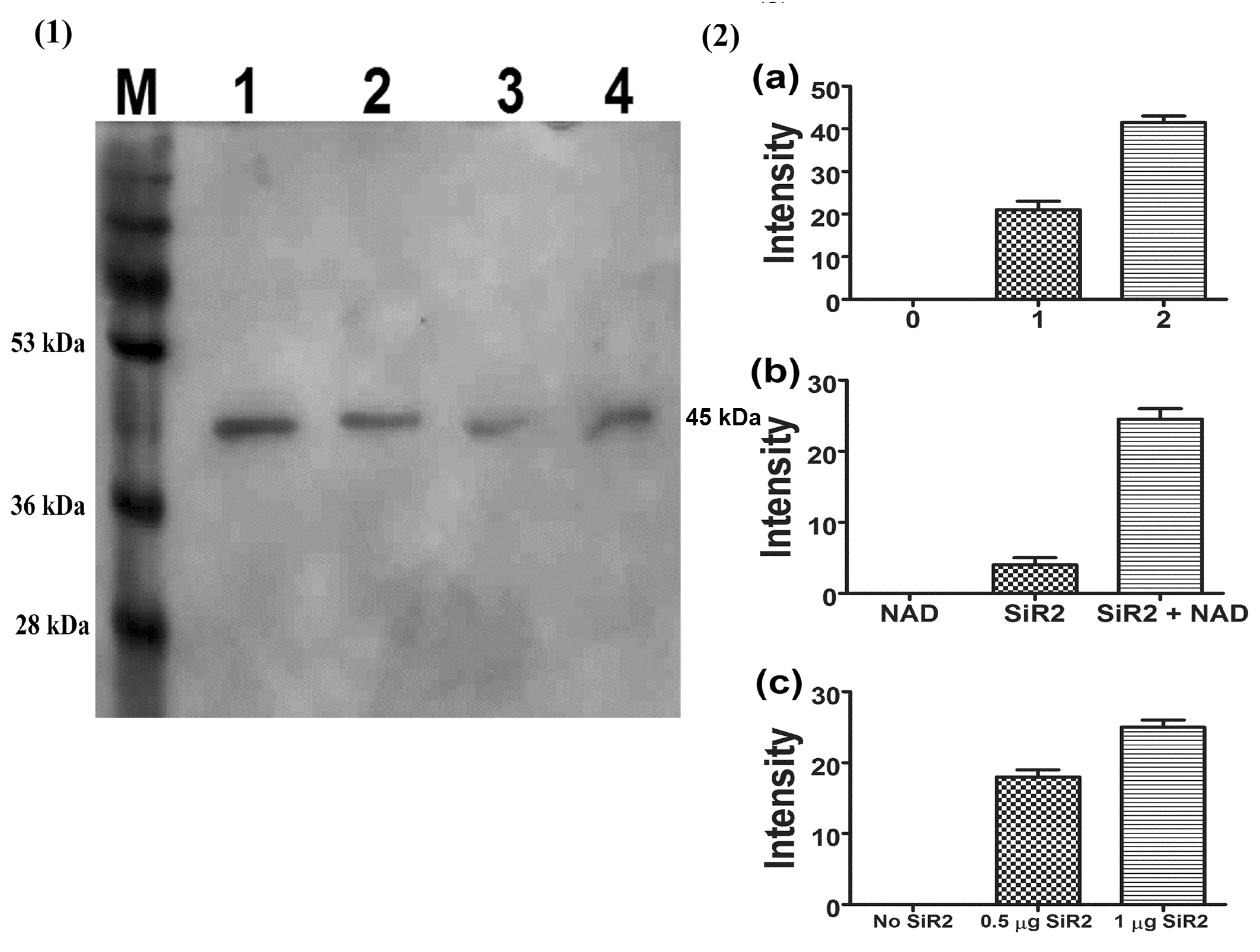

Supplement: S4 Fig — (1) SDS PAGE analysis of glutaraldehyde cross linked recombinant protein. M represents molecular marker. Lane1, 2, 3 and 4 represent 0, 0.5, 1 and 1.5% of glutaraldehyde. We observed single band at the position near 45 kDA with increasing concentration of glutaraldehyde. Similarly a single band was observed in the absence of glutaraldehyde as shown. (2) We have analyzed NAD depended deacetylase activity of recombinant SIR2 deacetylase. (S4a Fig) Represents substrate depended activity of recombinant protein, bar 0, 1 and 2 show 0, 1 and 2 micro molar concentration of substrate peptide in standard reaction. (S4b Fig) represents NAD depended activity. c. represents effect of increasing concentration on enzyme activity. (S4c Fig) Figures show the fluorescence measurement of enzyme reaction mixture. (TIF) [file pntd.0003557.s004.tif]
