# Supplementary material for: Recombinant NAD-dependent SIR-2 Protein of Leishmania donovani: Immunobiochemical Characterization as a Potential Vaccine against Visceral Leishmaniasis
Source: PLoS Negl Trop Dis. 2015 Mar 6;9(3):e0003557. doi: 10.1371/journal.pntd.0003557 (PMC4351947; doi:10.1371/journal.pntd.0003557)
Supplement: S1 Table — (DOC) [file pntd.0003557.s005.doc]

**Table S1 -** indicating predicted MHC-I epitope sequences, their length, position and method used for identification.

| Allele name | Start | End | Peptide length | Sequence | Method used |
| --- | --- | --- | --- | --- | --- |
| HLA-A*02:06 | 219 | 227 | 9 | MQVHPFALL | Consensus (ann,smm) |
| HLA-A*33:03 | 72 | 80 | 9 | DAFSLTLLR | Netmhcpan |
| HLA-A*33:03 | 324 | 332 | 9 | DVCRDVFFR | Netmhcpan |
| HLA-A*33:03 | 167 | 175 | 9 | EAMSGTVSR | Netmhcpan |
| HLA-A*11:01 | 225 | 233 | 9 | ALLPCVVPK | Consensus (ann,smm) |
| HLA-A*11:01 | 53 | 61 | 9 | SSDTGIYAK | Consensus (ann,smm) |
| HLA-A*33:03 | 229 | 237 | 9 | CVVPKSIPR | Netmhcpan |
