# Supplementary material for: Recombinant NAD-dependent SIR-2 Protein of Leishmania donovani: Immunobiochemical Characterization as a Potential Vaccine against Visceral Leishmaniasis
Source: PLoS Negl Trop Dis. 2015 Mar 6;9(3):e0003557. doi: 10.1371/journal.pntd.0003557 (PMC4351947; doi:10.1371/journal.pntd.0003557)
Supplement: S2 Table — (DOC) [file pntd.0003557.s006.doc]

**Table S2-** indicating predicted MHC-II epitope sequences, their length, position and method used for identification.

| Allele name | Start | End | Peptide length | Sequence | Method used |
| --- | --- | --- | --- | --- | --- |
| HLA-DRB1*0701 | 98 | 112 | 15 | GHFQPTAVHHFIRLL | Consensus (ann,smm) |
| HLA-DRB1*0701 | 97 | 111 | 15 | PGHFQPTAVHHFIRL | Consensus (comb.lib.,smm,nn) |
| HLA-DRB1*0701 | 95 | 109 | 15 | LWPGHFQPTAVHHFI | Consensus (comb.lib.,smm,nn) |
| HLA-DRB1*0701 | 96 | 110 | 15 | WPGHFQPTAVHHFIR | Consensus (comb.lib.,smm,nn) |
| HLA-DRB1*0701 | 94 | 108 | 15 | NLWPGHFQPTAVHHF | Consensus (comb.lib.,smm,nn) |
| HLA-DQA1*0102/ DQB1*0602 | 36 | 50 | 15 | VLVGAGASVAAGIPD | Consensus (comb.lib.,smm,nn) |
| HLA-DQA1*0102/ DQB1*0602 | 35 | 49 | 15 | LVLVGAGASVAAGIP | Consensus (comb.lib.,smm,nn) |
